# Characterization of isolates from two phase 3 uncomplicated urinary tract infection trials (EAGLE-2 and EAGLE-3) that show an apparent ≥4-fold increase in gepotidacin MIC between baseline and post-baseline visits

**DOI:** 10.1093/jacamr/dlag186

**Published:** 2026-09-01

**Authors:** Chase A Weikel, John Breton, Deborah Butler, Josh West, Amanda Sheets, John H Kimbrough, Mariana Castanheira, Rodrigo E Mendes, Nicole E Scangarella-Oman

**Affiliations:** Vaccines and Infectious Disease Research Unit, GSK, Collegeville, PA, USA; Vaccines and Infectious Disease Research Unit, GSK, Collegeville, PA, USA; Vaccines and Infectious Disease Research Unit, GSK, Collegeville, PA, USA; Vaccines and Infectious Disease Research Unit, GSK, Collegeville, PA, USA; Vaccines and Infectious Disease Research Unit, GSK, Collegeville, PA, USA; Element Iowa City (JMI Laboratories), North Liberty, IA, USA; Element Iowa City (JMI Laboratories), North Liberty, IA, USA; Element Iowa City (JMI Laboratories), North Liberty, IA, USA; Vaccines and Infectious Disease Research Unit, GSK, Collegeville, PA, USA

## Abstract

**Background and objectives:**

Effective therapies for the treatment of uncomplicated urinary tract infections (uUTIs) are increasingly limited due to rising multidrug resistance, necessitating the development of novel antimicrobials. Gepotidacin is a first-in-class triazaacenaphthylene antibiotic that inhibits bacterial DNA gyrase and topoisomerase IV through a distinct binding site, a unique mechanism of action, and with well-balanced inhibition for most pathogens. Gepotidacin was recently approved by the FDA and MHRA for the treatment of uUTIs and by the FDA for uncomplicated urogenital gonorrhoea. This study investigated the development of resistance to gepotidacin among isolates from two Phase 3 uUTI clinical trials (EAGLE-2 [NCT04020341]/EAGLE-3 [NCT04187144]).

**Methods:**

Post-baseline isolates exhibiting a reproducible ≥4-fold increase in minimum inhibitory concentration (MIC) and non-distinct multi-locus sequence typing/pulsed-field gel electrophoresis results underwent single nucleotide polymorphism/insertion-deletion analysis.

**Results:**

Across both studies, initial MIC testing showed 58/1045 (5.6%) patients with pathogen(s) demonstrating an apparent ≥4-fold increase in post-baseline gepotidacin MIC. Analyses identified only one patient with multiple *Escherichia coli* isolate pairs displaying potential development of resistance to gepotidacin. While the post-baseline isolates did not meet the putative direct descendancy criteria, we have conservatively considered this a case of possible development of resistance due to the rarity of the gepotidacin target-specific mutation (ParC D79G) in the on-therapy and test-of-cure *E. coli* isolates.

**Conclusions:**

Collectively, results from the Phase 3 trials indicate a low incidence of resistance development to gepotidacin, with only one potential case among gepotidacin-treated patients (1/1045; 0.096%). Further research is warranted to elucidate the mechanisms underlying elevated post-baseline gepotidacin MICs.

## Introduction

Urinary tract infections (UTIs) are among the most common bacterial outpatient infections, with a lifetime incidence of 50%–60% in adult females (≥18 years of age),^1^ and an estimated 11% experiencing at least one episode annually, with the majority of the annual cases occurring among females who have experienced two or more UTI episodes in their lifetime.^2^ The incidence generally increases with age, apart from a spike in young, sexually active women.^3–5^ The predominant uropathogen in community-acquired UTIs is *Escherichia coli*, accounting for approximately 75% of cases.^6–8^ Other uropathogens, including *Staphylococcus*, *Enterococcus*, *Klebsiella*, and *Proteus* species, collectively account for about 20% of cases.^6,8^ According to regulatory guidelines for registrational trials, uncomplicated UTIs (uUTIs) occur in female patients without known urological abnormalities or complicating comorbidities and not accompanied by signs of systemic infection,^9^ although recently updated clinical practice guidelines expand this definition to include infections confined to the bladder in both female and male patients and regardless of urologic abnormalities, immune status, or diabetes.^10^ Existing oral therapies for uUTI are increasingly limited due to rising trends in multidrug-resistant (MDR) pathogens, including extended-spectrum beta-lactamase-producing Enterobacterales, which often show cross-resistance to other current treatments. This has created an urgent need for new therapeutic agents with activity against MDR uropathogens and a low propensity for collateral damage.^11–14^

Gepotidacin is a novel, bactericidal, first-in-class triazaacenaphthylene antibiotic that inhibits bacterial DNA replication through a distinct binding site, and a unique mechanism of action^15,16^ which provides activity against most target uUTI uropathogens including antibiotic-resistant isolates.^17,18^ Due to its well-balanced (for most uropathogens) inhibition of two enzymes (DNA gyrase and topoisomerase IV), a gepotidacin target-specific mutation in a single enzyme does not significantly affect gepotidacin susceptibility.^19,20^ Based on the results of Phase 3 uUTI studies, EAGLE-2 and EAGLE-3 (NCT04020341 and NCT04187144), gepotidacin was approved in March 2025 by the U.S. Food and Drug Administration (FDA) for the treatment of female adult and paediatric patients 12 years of age and older weighing at least 40 kg with uUTIs caused by the following susceptible microorganisms: *Escherichia coli*, *Klebsiella pneumoniae*, *Citrobacter freundii* complex, *Staphylococcus saprophyticus* and *Enterococcus faecalis*.^21,22^ Gepotidacin was then approved in August 2025 by the Medicines and Healthcare products Regulatory Agency (MHRA) for the treatment of uncomplicated urinary tract infections.^23^ Further, FDA approval was obtained in December 2025 for the oral treatment of uncomplicated urogenital gonorrhoea in adult and paediatric patients 12 years of age and older.^22^

During the EAGLE-2 and EAGLE-3 Phase 3 clinical trials, ≥4-fold increases in the assigned study treatment minimum inhibitory concentration (MIC) for a given post-baseline isolate (i.e. on-therapy [OT], test-of-cure [TOC] or follow-up [FU]) when compared with the MIC for the respective baseline isolate of the same genus and species, regardless of genetic relatedness, were monitored. The primary purpose of this study was to analyse isolate pairs in the intent-to-treat (ITT) population with an apparent ≥4-fold increase in post-baseline gepotidacin MIC compared to baseline MIC and non-distinct multi-locus sequence type/pulsed-field gel electrophoresis results (MLST/PFGE) to assess whether the post-baseline isolates were a direct descendant from the respective baseline isolate and identify the associated gepotidacin resistance mechanisms.

## Materials and methods

### Use of generative AI in manuscript preparation

Generative artificial intelligence (GenAI) was used during the drafting of this manuscript. Specifically, GenAI was utilized in collating and streamlining parts of the introduction and abstract.

### Ethics

The trials were conducted in accordance with the Declaration of Helsinki principles and the International Council for Harmonization guidelines for good clinical practice. The protocols were approved by all necessary ethics committees and institutional review boards. All participants’ written consent was obtained.

### Clinical trial uropathogens

Mid-stream clean-catch urine samples were collected from all patients (non-pregnant females [≥12 years] with uUTI symptoms) at all visits (baseline and post-baseline [i.e. OT, TOC or FU]). A portion of the urine sample was aseptically transferred to a Becton–Dickinson grey-top urine culture and sensitivity Vacutainer^™^ tube and shipped in refrigerated conditions to the central laboratory (Pharmaceutical Product Development's Global Central Laboratory [PPD GCL], Highland Heights, Kentucky, USA). Samples were processed for culture, isolate identification, antimicrobial susceptibility testing (AST), and epidemiological typing according to Clinical and Laboratory Standards Institute (CLSI) guidelines.^21,24,25^

### Antimicrobial susceptibility testing and epidemiological typing

Initial AST was conducted by the central laboratory (PPD GCL) using custom-dried microdilution panels (Thermo Fisher Scientific). For patients with isolates of the same species recovered from multiple study visits, isolates were initially epidemiologically typed through MLST, when typing schema were available, using the MLST database (PubMLST) available at https://pubmlst.org. For isolates in which one or more alleles deviated from established STs, ‘-like’ was appended to the nearest matching MLST scheme. The relationships of *Proteus mirabilis* isolates were determined by PFGE using standard and pre-established protocols.^26^ Gel pattern analysis of post-baseline strains was compared to those of their respective baseline strains using the GelCompar II software (Applied Math, Kortrijk, Belgium), and each post-baseline strain received a similarity coefficient (%) value from a comparison to its respective baseline isolate. Isolates with similarity coefficients <85% were considered unrelated.^27^

### MIC confirmation and genetic characterization of uropathogens

Baseline and post-baseline isolate pairs demonstrating an apparent ≥4-fold increase in gepotidacin MIC and demonstrating non-distinct MLST/PFGE results were processed by a third-party laboratory (Element, Iowa City/formerly JMI Laboratories, North Liberty, Iowa, USA) for MIC confirmation via broth microdilution (BMD) according to CLSI guidelines.^24^ The 96-well microdilution panels were manufactured by Element, Iowa City. Isolates were tested in technical triplicate using the same inoculum.

All isolates demonstrating a reproducible ≥4-fold increase between baseline/post-baseline gepotidacin MICs and non-distinct MLST/PFGEs results underwent molecular characterization (single nucleotide polymorphism [SNP]/insertion-deletion [indel] analysis) to assess genetic relatedness and the potential development of resistance.

### DNA extraction and genome sequencing

Total genomic DNA was extracted and purified using the KingFisher Cell and Tissue DNA kit (Thermo Fisher Scientific, Waltham, Massachusetts, USA) or the Applied Biosystems MagMax Multi-Sample Ultra 2.0 Kit (Thermo Fisher Scientific) in a robotic KingFisher^™^ Flex Magnetic Particle Processor (Thermo Fisher Scientific). Total genomic DNA was used as input material for library construction. DNA libraries were prepared using the Nextera XT^™^ library construction protocol and index kit (Illumina, San Diego, California, USA) or the Illumina DNA Prep library construction protocol and index kit (Illumina) and sequenced on a MiSeq Sequencer (Illumina) using a MiSeq Reagent Kit v3 (600 cycle) or a NextSeq 1000 Sequencer using a NextSeq 1000/2000 P2 v3 (300 cycle) reagent cartridge and a NextSeq 1000/2000 P2 flow cell (Illumina). FASTQ format files for each sample set were assembled independently using *de novo* assembler SPAdes 3.11.1.^28^

High molecular weight DNA was obtained using the Nanobind CBB Big DNA Kit (PacBio, Menlo Park, California, USA) according to the manufacturer's instructions. Purified genomic DNA was allowed to solubilize at room temperature for 16 h. Purity was assessed using a NanoDrop 2000 spectrophotometer (Thermo Fisher Scientific) and DNA concentration was measured using the Invitrogen Qubit 1 ×  dsDNA HS Assay Kit (Thermo Fisher Scientific) on a Life Technologies Qubit 3.0 Fluorometer (Thermo Fisher Scientific). Sequencing library preparation was carried out with Rapid Barcoding Kit 24 V14 Sequencing Kit (SQK-RBK-114.24) using ∼100 ng input DNA per sample on an R10.4.1 flow cell using the MinION Mk1C sequencer controlled by MinKNOW version 5.3.6 (Oxford Nanopore Technologies Ltd, Oxford, UK). Library preparation and sequencing were performed according to the manufacturer's protocol. Basecalling was performed with Guppy version 6.4.2.

### Screening for plasmid and QRDR-mediated resistance mechanisms

Isolates were screened against a curated database of plasmid-mediated quinolone resistance (PMQR) genes. Sequences displaying 100% identity to reference sequences were named accordingly and genes with <100% identity were named with the suffix ‘-like’ relative to the named gene with the highest percent identity. Additionally, the sequences associated with the quinolone resistance-determining region (QRDR) of *gyrA* and *gyrB*, encoding subunits of DNA gyrase, and *parC* and *parE* (encoding subunits of topoisomerase IV) were also examined. These QRDR sequences were compared to those from susceptible strains *E. coli* ATCC 25922 or *K. pneumoniae* ATCC 13883 to detect mutations potentially associated with fluoroquinolone resistance.^29^

### Assessment of genetic relatedness

To understand the genetic relationships between baseline and post-baseline isolates from the same patient, hybrid assemblies of short- and long-read sequencing were created using Unicycler v0.4.9-beta.^30^ Alterations were identified using MAUVE V2.4.0^28,31^ and confirmed by mapping quality-trimmed reads independent of the baseline assembly. Reads were mapped using BWA v0.7.15-r1140.^32^ Indel sites were realigned using IndelRealigner from the GATK toolbox v3.8-1-0gf15c13ef.^33^ High-confidence variants were called by SAMtools v1.8 and filtered by BCFtools v1.8.^32,34,35^ Filtering criteria for the variant call format file were a minimum read depth of 4 (≥2 reads per strand), >30 map quality, >50 phred-scaled variant quality, no significant strand bias, and >75% of mutations within reads to support the presence of any given alteration. Repeat regions of > 50 bp were identified with MUMmer v3.1 and excluded from analysis.^36,37^

Baseline assembly was annotated using Prokka v1.14.5 and alterations in post-baseline isolates were annotated using Snpeff 4.3t.^38,39^ Short reads were subjected to quality trimming using a sliding window threshold of Q18. Additional analysis was performed by nucDiff to capture all indels and uncovered regions.^40^ Uncovered regions were identified as stretches of nucleotide sequences missing from the comparator when matched with the reference sequence. Average mapping quality and phred-scaled variant quality scores were collected from the alignment of Illumina short reads to the hybrid-assembled parent genomes, as described above. For all reported variations from the parental strain, the average read-mapping quality was 60 (MAPQ = 60), and the phred-scaled variant probability was at least 205. Both metrics indicate high confidence in the reported variants. The majority of bases that supported alterations showed high base-call confidence, with an average percentage of 86  ±  8.0% of bases supporting an alteration with quality ≥Q30.

Genetic relatedness (i.e. putative direct descendancy) of post-baseline *E. coli* and *K. pneumoniae* uropathogens from the respective baseline strain was assessed based on the total number of genetic alterations (i.e. SNPs and indels <25 bp) accumulated between the baseline and post-baseline visit time frame. For all comparisons, any post-baseline isolates recovered during or after exposure to study treatment were compared solely to the isolate recovered during the pre-treatment baseline visit (e.g. OT versus baseline, TOC versus baseline, etc.). The number of accumulated alterations for determination of potential development of resistance was set at  ≤ 1 for isolates collected ≤5 days after the baseline visit, ≤2 for isolates collected between 6 and 14 days after the baseline visit, and ≤5 for isolates collected between 15 and 30 days after the baseline visit, consistent with previous reports for *in vivo E. coli* and *K. pneumoniae* evolutionary rates (Table 3).^41–44^

## Results

### Analyses of apparent *≥*4-fold increases in gepotidacin MIC

**Figure 1.**
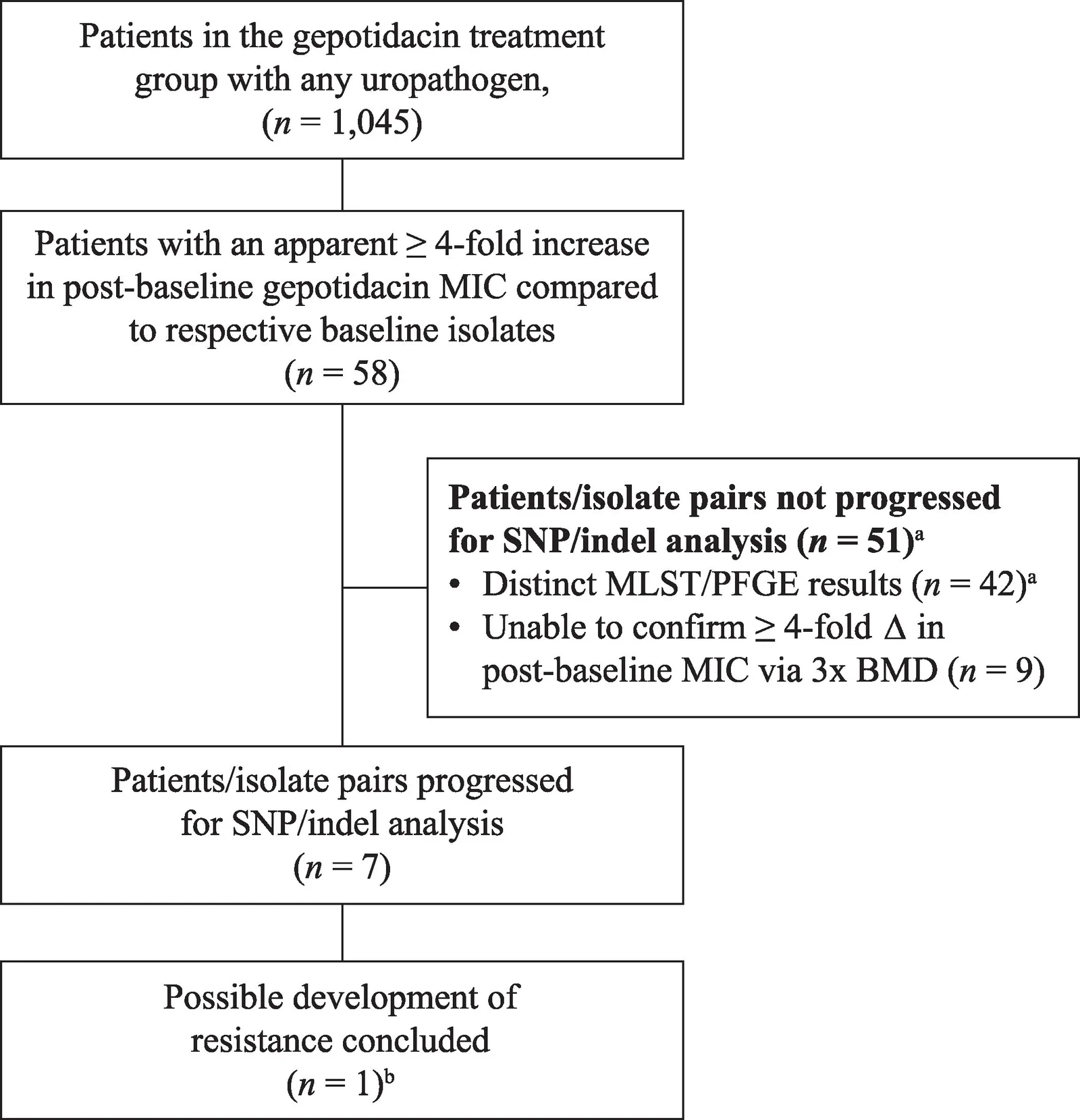
Evaluation of potential development of resistance to gepotidacin during EAGLE-2 and EAGLE-3 (pooled ITT population). ^a^When referring to the number of patients, *n* = 51/*n* = 42; when referring to the number of isolate pairs, *n* = 52/*n* = 43 as patient #1 possessed two distinct pairs of *E. coli* with unique morphologies at baseline each with an apparent ≥4-fold increase in post-baseline gepotidacin MIC. One isolate pair had distinct MLSTs while the other pair was progressed for SNP/indel analysis. ^b^While post-baseline isolates from one patient (#7) did not meet the SNP criteria to have originated from the baseline isolate, this is conservatively considered to be possible development of resistance due to the ParC D79G mutation. BMD, broth microdilution; ITT, intent-to-treat; MIC, minimum inhibitory concentration; MLST/PFGE, multi-locus sequence type/pulsed-field gel electrophoresis; SNP/indel, single nucleotide polymorphism/insertion-deletion.

In the ITT population from the pooled EAGLE-2 and EAGLE-3 studies, 5.6% (58/1045) of patients in the gepotidacin treatment group and 1.9% (19/1011] of patients in the nitrofurantoin group had post-baseline isolates with an apparent ≥4-fold increase in study treatment MIC compared to respective baseline isolates (Table 1). In the gepotidacin group, MLST/PFGE analysis showed that isolate pairs from 42 patients were genetically distinct. Further, triplicate BMD MIC testing did not confirm the initially observed ≥4-fold increase in MIC for post-baseline isolates from 9 additional patients (Figure 1).

**Table 1.** Summary of reduction in susceptibility to study treatment (ITT population)

Uropathogen/visit	EAGLE 2		EAGLE 3		Pooled EAGLE 2 & EAGLE 3	
	Gepotidacin^a^ 1500 mg BID (*N* = 767)	Nitrofurantoin^b^ 100 mg BID (*N* = 764)	Gepotidacin^a^ 1500 mg BID (*N* = 805)	Nitrofurantoin^b^ 100 mg BID (*N* = 800)	Gepotidacin^a^ 1500 mg BID (*N* = 1572)	Nitrofurantoin^b^ 100 mg BID (*N* = 1564)
Any uropathogen	547	519	498	492	1045	1011
OT	11 (2.0%)	5 (1.0%)	2 (0.4%)	3 (0.6%)	13 (1.2%)	8 (0.8%)
TOC	18 (3.3%)	3 (0.6%)	1 (0.2%)	2 (0.4%)	19 (1.8%)	5 (0.5%)
FU	27 (4.9%)	5 (1.0%)	6 (1.2%)	3 (0.6%)	33 (3.2%)	8 (0.8%)
Any time post-baseline	49 (9.0%)	12 (2.3%)	9 (1.8%)	7 (1.4%)	58 (5.6%)	19 (1.9%)

^a^Data represents summary of reduction in susceptibility to gepotidacin in the gepotidacin treatment group.

^b^Data represents summary of reduction in susceptibility to nitrofurantoin in the nitrofurantoin treatment group.

Percentage is calculated using the number of subjects at a given visit with ‘Any uropathogen (*n*)’ (≥10^3^ µg/mL) as the denominator.

Reduction in susceptibility is defined as post-baseline uropathogens with an apparent ≥4-fold increase in MIC when compared with the MIC at baseline of a uropathogen of the same species from the same subject.

BID, twice daily orally; ITT, intent-to-treat; MIC, minimum inhibitory concentration in µg/mL; OT, on-therapy; TOC, test-of-cure; FU, follow-up.

Therefore, isolate pairs (*E. coli*, *n* = 4; *K. pneumoniae*, *n* = 3) from the remaining seven patients (7/1045; 0.7%) were progressed for SNP/indel analysis as they showed identical MLST/PFGE and a reproducible ≥4-fold increase in gepotidacin MIC for the post-baseline isolates (Table 2).

**Table 2.** SNP/indel analysis results of isolate pairs with reproducible ≥4-fold increase in gepotidacin MIC and non-distinct MLST results from gepotidacin treatment group

Patient number	Organism	Visit	Days post-baseline	GEP MIC (µg/mL)	Fluoroquinolone resistance mechanism		SNP/indel analysis	
					QRDR^a^	Other	Number	Key variations^b^
**1**	*E. coli*	Baseline	N/A	8	S83L, D87N/S80I/WT	QnrS1		
		OT	4	64	S83L, D87N/S80I/WT	QnrS1	13	AcrB^S288G G570C^
**2**	*E. coli*	Baseline	N/A	4	WT/WT/WT	QnrB19		
		FU	29	32	WT/WT/WT	QnrS1	12 492	QnrS1
**3**	*K. pneumoniae*	Baseline	N/A	16	S83I/WT/S80I/WT	aac(6′)-Ib-cr, OqxA11, OqxB19, QnrB1		
		TOC	9	>64	S83I/WT/S80I/WT	aac(6′)-Ib-cr, OqxA11, OqxB19, QnrB1	9	None det.
**4**	*E. coli*	Baseline	N/A	0.12	S83L, D87N/S80I/L416F	None det.		
		FU	30	0.5	S83L, D87N/S80I/L416F	None det.	18	MdfA^G10R^
**5**	*K. pneumoniae*	Baseline	N/A	8	WT/WT/WT	OqxA^G26D^/OqxB14		
		TOC	10	32	WT/WT/WT	QnrS1/OqxA^G26D^/OqxB14	3	QnrS1
**6**	*K. pneumoniae*	Baseline	N/A	4	WT/WT/WT	OqxA/OqxB19^A551V V562M^		
		OT	3	32	WT/WT/WT	OqxA/OqxB19^A551V V562M^	2	OqxR^ΔY109^
**7**	*E. coli*	Baseline	N/A	16	WT/WT/WT	QnrS1/QnrB19		
		OT	1	128	WT/D79G/WT	QnrS1/QnrB19	2	ParC^D79G^ MFS^P372S^
		TOC	9	128	WT/D79G/WT	QnrS1/QnrB19	4	ParC^D79G^ MFS^P372S^ AcrB^G288A^
		Day 34^c^	33	128	WT/D79G/WT	QnrS1/QnrB19	4	ParC^D79G^ MFS^P372S^ AcrB^A279T^

^a^Presented as *gyrA*/ParC/ParE; variations were not detected in gyrB.

^b^These variations are thought to be possible putative resistance mechanisms.

^c^Isolate was recovered outside of FU analysis visit window (Days 21–31).

FU, follow-up; GEP, gepotidacin; indel, insertion-deletion; MFS, major facilitator superfamily; MIC, minimum inhibitory concentration in µg/mL; MLST, multi-locus sequence type; N/A, not applicable; None det., none detected in the respective column/category; OT, on-therapy; QRDR, quinolone resistance determining region; SNP, single nucleotide polymorphism; TOC, test-of-cure; WT, wild-type.

### SNP/indel analysis results for the 7 isolate pairs

SNP/indel analysis of the 7 baseline/post-baseline isolate pairs did not suggest possible development of resistance to gepotidacin due to SNP accumulation exceeding that expected for the timeframe (Table 2 and Table 3).

**Table 3.** Genetic relatedness (putative direct descendancy) criteria

Days post-baseline visit	Max number of accumulated SNP/indel alterations
≤5	≤1
6–14	≤2
15–30	≤5

indel, insertion-deletion; SNP, single nucleotide polymorphism.

However, patient #7 had post-baseline *E. coli* isolates recovered at OT, TOC, and at an unscheduled visit on Day 34 (occurring 1, 9 and 33 days post-baseline), in which 2, 4 and 4 SNPs were identified relative to the baseline isolate (Table 2 and Table S1 (available as Supplementary data at *JAC-AMR* Online)). All three post-baseline isolates had a D79G variation in the ParC subunit of topoisomerase IV and a P372S variation in a gene encoding a putative major facilitator superfamily protein. Post-baseline isolates cultured at TOC and on Day 34 also had unique variations in the AcrB efflux pump component (G288A and A279T, respectively). Broth microdilution testing revealed that the gepotidacin MIC for the post-baseline isolates (128 µg/mL) were 8-fold higher than the MIC for the baseline isolate (16 µg/mL) (Table 2 and Table S1). While SNP/indel analysis for patient #7 did not suggest possible development of resistance to gepotidacin occurring between the baseline and OT or TOC isolates due to SNP accumulation exceeding that expected for the timeframe (Table 3), a conservative stance was taken to consider this as possible development of resistance, due to the gepotidacin target-specific ParC D79G mutation at OT and TOC. Furthermore, while the Day 34 isolate was collected outside of the FU window (Day 21–31), the number of SNPs observed aligns with the defined relatedness threshold (<5 SNPs 33 days from baseline; Table 2).

## Discussion

In the EAGLE-2 and EAGLE-3 studies, a total of 58 patients (5.6% of 1045 patients with any uropathogen) in the gepotidacin treatment group from the pooled ITT population had baseline/post-baseline isolate pairs of the same genus and species, with an apparent ≥4-fold decrease in susceptibility to gepotidacin. Of the 58 patients from the gepotidacin treatment group, 49 were from EAGLE-2 and 9 were from EAGLE-3. The aetiology of this difference between the studies is unknown.

To consider a  ≥ 4-fold increase in post-baseline gepotidacin MIC as development of resistance, the post-baseline isolate(s) are required to have originated from the baseline strain (i.e. possess identical MLST/PFGEs and SNP/indel accumulation within limits for the expected timeframe). The majority of suspected ≥4-fold increases in gepotidacin MIC could not be reproduced with repeat triplicate MIC testing or the isolates were found not to be genetically related via MLST/PFGE typing or SNP/indel analysis and, thus, not considered cases of development of resistance. Possible explanations for the higher post-baseline gepotidacin MIC in the patients in which there was no confirmed development of resistance include the possibility that baseline/post-baseline isolate pairs represent cases of colonization, reinfection with a unique isolate, or coinfection with a unique isolate that was not recovered from the baseline culture (e.g. subpopulation undetectable by culture).

Our investigation did, however, identify a possible case of development of resistance to gepotidacin in *E. coli* from one patient. The SNP/indel analysis for the *E. coli* isolate pairs from patient #7 did not directly suggest development of resistance to gepotidacin between the baseline and OT or TOC isolates (i.e.  >1 SNP observed within 24 h from baseline), but it did show a rare gepotidacin target-specific mutation (ParC D79G) in the post-baseline isolates. And while a study with isogenic *E. coli* and *K. pneumoniae* mutants has shown that a single target mutation at ParC D79 or GyrA D82 has limited impact on gepotidacin MICs,^19^ we have conservatively noted this as a possible development of resistance and acknowledge that an unknown resistance factor, such as overexpression of efflux, may be potentially contributing to the increase in gepotidacin MIC (128 µg/mL; resistant to gepotidacin per FDA breakpoints).^45^ Isogenic studies are required to define the mechanisms responsible for the elevated gepotidacin MICs observed for the post-baseline isolates recovered from patient #7. Of note, despite the categorical change in susceptibility between the baseline and post-baseline visits, this patient reported complete resolution of their urinary symptoms and did not require further non-study antibiotic treatment, suggesting this case of potential development of resistance was not clinically significant (Table S1).

It's also worth noting that all but one of the post-baseline isolates with increased gepotidacin MICs presented with either *qnr* acquisition(s) or alterations within *oqxR* (Table 2). However, prior non-clinical studies report a wide range of gepotidacin MICs (0.25–64 µg/mL) against *E. coli* and *K. pneumoniae* isolates harbouring PMQR genes.^46–48^ Further, studies involving isogenic efflux knockout strains confirm that efflux plays a part in gepotidacin MICs (data not published), but no single genotype/phenotype or combination thereof has been definitively linked to higher gepotidacin MICs. Further analysis is needed to assess the extent to which these mechanisms impact gepotidacin susceptibility.

Given the scope of this study and the focus on assessing the development of resistance to gepotidacin during the EAGLE-2 and EAGLE-3 studies, limited testing was conducted for isolate pairs with an apparent ≥4-fold increase in nitrofurantoin MICs between baseline and post-baseline isolates. However, we do acknowledge the initial rate of reduced susceptibility to study treatment was lower in the nitrofurantoin arm (19 patients [1.9% of 1011]). Because additional testing of isolates from the nitrofurantoin arm was limited, the relevance of the difference between rates of reduced susceptibility between treatment arms is unknown. However, the higher rate of apparent reduced susceptibility in the gepotidacin group compared with the nitrofurantoin group was not associated with a higher overall rate of rescue antibiotic use in the study, with 4.7% (74/1572) in the gepotidacin group versus 7.0% (109/1564) in the nitrofurantoin group.^21^ Lastly, we acknowledge that the conclusions drawn from our analysis are limited by the size of the dataset. Although EAGLE-2 and EAGLE-3 are among the largest registrational trials for uUTI, providing a robust clinical dataset for assessing the risk of resistance development following gepotidacin treatment in the context of a controlled trial, real-world susceptibility and development of resistance to gepotidacin at a population level will need to be monitored through future surveillance studies.

Overall, the results of this study are consistent with prior hollow-fibre *in vitro* infection models, frequency of spontaneous resistance and isogenic data that predicted the low potential for development of resistance to gepotidacin during treatment.^49,50^ Further, it has been shown that gepotidacin target-specific mutations in both target enzymes are needed to significantly reduce gepotidacin susceptibility.^20^ We found only one case of possible development of resistance (*E. coli*; patient #7) to gepotidacin occurred in the gepotidacin Phase 3 uUTI studies, EAGLE-2 and EAGLE-3. This low prevalence of resistance to gepotidacin further enhances its viability as a novel therapeutic option for the treatment of patients with uUTI.

## Supplementary Material

dlag186_Supplementary_Data

## Supplementary data

Table S1 is available as Supplementary data at *JAC-AMR* Online.

## References

[1] Medina M, Castillo-Pino E. An introduction to the epidemiology and burden of urinary tract infections. Ther Adv Urol 2019; 11: 1756287219832172. 10.1177/1756287219832172 31105774 PMC6502976

[2] Foxman B, Barlow R, D'Arcy H et al Urinary tract infection: self-reported incidence and associated costs. Ann Epidemiol 2000; 10: 509–15. 10.1016/s1047-2797(00)00072-7 11118930

[3] Fihn SD . Clinical practice. Acute uncomplicated urinary tract infection in women. N Engl J Med 2003; 349: 259–66. 10.1056/NEJMcp030027 12867610

[4] Schmiemann G, Kniehl E, Gebhardt K et al The diagnosis of urinary tract infection: a systematic review. Dtsch Arztebl Int 2010; 107: 361–7. 10.3238/arztebl.2010.0361 20539810 PMC2883276

[5] Suskind AM, Saigal CS, Hanley JM et al Incidence and management of uncomplicated recurrent urinary tract infections in a national sample of women in the United States. Urology 2016; 90: 50–5. 10.1016/j.urology.2015.11.051 26825489 PMC4822518

[6] Foxman B . The epidemiology of urinary tract infection. Nat Rev Urol 2010; 7: 653–60. 10.1038/nrurol.2010.190 21139641

[7] Foxman B . Urinary tract infection syndromes: occurrence, recurrence, bacteriology, risk factors, and disease burden. Infect Dis Clin North Am 2014; 28: 1–13. 10.1016/j.idc.2013.09.003 24484571

[8] Flores-Mireles AL, Walker JN, Caparon M et al Urinary tract infections: epidemiology, mechanisms of infection and treatment options. Nat Rev Microbiol 2015; 13: 269–84. 10.1038/nrmicro3432 25853778 PMC4457377

[9] U.S. Food and Drug Administration . *Guidance for industry uncomplicated urinary tract infections: Developing drugs for treatment*. 2019. https://www.fda.gov/media/129531/download

[10] Trautner BW, Cortes-Penfield NW, Gupta K et al Clinical practice guideline by Infectious Diseases Society of America (IDSA): 2025 guideline on management and treatment of complicated urinary tract infections: selection of antibiotic therapy for complicated UTI. Clin Infect Dis 2025; **82**: i36–67. 10.1093/cid/ciaf460 41419213

[11] Spellberg B, Doi Y. The rise of fluoroquinolone-resistant *Escherichia coli* in the community: scarier than we thought. J Infect Dis 2015; 212: 1853–5. 10.1093/infdis/jiv279 25969562 PMC4655852

[12] Abadi A, Rizvanov A, Haertle T et al World Health Organization report: current crisis of antibiotic resistance. BioNanoScience 2019; 9: 778–88. 10.1007/s12668-019-00658-4

[13] Centers for Disease Control and Prevention . *Antibiotic resistance threats in the United States*. 2019. https://stacks.cdc.gov/view/cdc/82532

[14] European Centre for Disease Prevention and Control . *Surveillance of antimicrobial resistance in Europe 2022*. 2023. https://www.ecdc.europa.eu/en/publications-data/surveillance-antimicrobial-resistance-europe-2022.

[15] Bax BD, Chan PF, Eggleston DS et al Type IIA topoisomerase inhibition by a new class of antibacterial agents. Nature 2010; 466: 935–40. 10.1038/nature09197 20686482

[16] Gibson EG, Bax B, Chan PF et al Mechanistic and structural basis for the actions of the antibacterial gepotidacin against *Staphylococcus aureus* gyrase. ACS Infect Dis 2019; 5: 570–81. 10.1021/acsinfecdis.8b00315 30757898 PMC6461504

[17] Mushtaq S, Vickers A, Sadouki Z et al In vitro activities of gepotidacin, a novel triazaacenaphthylene and topoisomerase IV DNA gyrase inhibitor, against gram-negative bacteria and Staphylococcus saprophyticus, poster p1849. 29th European Congress of Clinical Microbiology and Infectious Diseases (ECCMID), Amsterdam, The Netherlands, 2019. European Society of Clinical Microbiology and Infectious Diseases (ESCMID).

[18] Arends SJR, Butler D, Scangarella-Oman N et al Antimicrobial activity of gepotidacin tested against *Escherichia coli* and *Staphylococcus saprophyticus* isolates causing urinary tract infections in Medical Centers Worldwide (2019 to 2020). Antimicrob Agents Chemother 2023; 67: e0152522. 10.1128/aac.01525-22 36877017 PMC10112209

[19] Szili P, Draskovits G, Révész T et al Rapid evolution of reduced susceptibility against a balanced dual-targeting antibiotic through stepping-stone mutations. Antimicrob Agents Chemother 2019; 63:10.1128/aac.00207-19. 10.1128/aac.00207-19 PMC6709476 31235632

[20] Oviatt AA, Gibson EG, Huang J et al Interactions between gepotidacin and *Escherichia coli* gyrase and topoisomerase IV: genetic and biochemical evidence for well-balanced dual-targeting. ACS Infect Dis 2024; 10: 1137–51. 10.1021/acsinfecdis.3c00346 38606465 PMC11015057

[21] Wagenlehner F, Perry CR, Hooton TM et al Oral gepotidacin versus nitrofurantoin in patients with uncomplicated urinary tract infection (EAGLE-2 and EAGLE-3): two randomised, controlled, double-blind, double-dummy, Phase 3, non-inferiority trials. Lancet 2024; 403: 741–55. 10.1016/s0140-6736(23)02196-7 38342126

[22] GSK . *Blujepa (gepotidacin) [package insert]*. 2025. https://www.accessdata.fda.gov/drugsatfda_docs/label/2025/218230s001lbl.pdf

[23] GOV.UK . *Press release: MHRA approves UK’s first new type of antibiotic for urinary tract infections in nearly 30 years*. 2025. https://www.gov.uk/government/news/mhra-approves-uks-first-new-type-of-antibiotic-for-urinary-tract-infections-in-nearly-30-years

[24] CLSI . *Methods for dilution antimicrobial susceptibility tests for bacteria that grow aerobically, 11th ed. M07*. Clinical and Laboratory Standards Institute, 2018.

[25] CLSI . *Performance standards for antimicrobial susceptibility testing, 35th ed. M100*. Clinical and Laboratory Standards Institute, 2025. 10.1128/jcm.01623-23 PMC12421849 40772786

[26] Castanheira M, Deshpande LM, Costello A et al Epidemiology and carbapenem resistance mechanisms of carbapenem-non-susceptible *Pseudomonas aeruginosa* collected during 2009-11 in 14 European and Mediterranean countries. J Antimicrob Chemother 2014; 69: 1804–14. 10.1093/jac/dku048 24603963

[27] Tenover FC, Arbeit RD, Goering RV et al Interpreting chromosomal DNA restriction patterns produced by pulsed-field gel electrophoresis: criteria for bacterial strain typing. J Clin Microbiol 1995; 33: 2233–9. 10.1128/jcm.33.9.2233-2239.1995 7494007 PMC228385

[28] Bankevich A, Nurk S, Antipov D et al SPAdes: a new genome assembly algorithm and its applications to single-cell sequencing. J Comput Biol 2012; 19: 455–77. 10.1089/cmb.2012.0021 22506599 PMC3342519

[29] Mendes RE, Jones RN, Woosley LN et al Application of next-generation sequencing for characterization of surveillance and clinical trial isolates: analysis of the distribution of β-lactamase resistance genes and lineage background in the United States. Open Forum Infect Dis 2019; 6: S69–78. 10.1093/ofid/ofz004 30895217 PMC6419912

[30] Wick RR, Judd LM, Gorrie CL et al Unicycler: resolving bacterial genome assemblies from short and long sequencing reads. PLoS Comput Biol 2017; 13: e1005595. 10.1371/journal.pcbi.1005595 28594827 PMC5481147

[31] Darling AC, Mau B, Blattner FR et al Mauve: multiple alignment of conserved genomic sequence with rearrangements. Genome Res 2004; 14: 1394–403. 10.1101/gr.2289704 15231754 PMC442156

[32] Li H, Handsaker B, Wysoker A et al The sequence alignment/map format and SAMtools. Bioinformatics 2009; 25: 2078–9. 10.1093/bioinformatics/btp352 19505943 PMC2723002

[33] McKenna A, Hanna M, Banks E et al The Genome Analysis Toolkit: a MapReduce framework for analyzing next-generation DNA sequencing data. Genome Res 2010; 20: 1297–303. 10.1101/gr.107524.110 20644199 PMC2928508

[34] Narasimhan V, Danecek P, Scally A et al BCFtools/RoH: a hidden Markov model approach for detecting autozygosity from next-generation sequencing data. Bioinformatics 2016; 32: 1749–51. 10.1093/bioinformatics/btw044 26826718 PMC4892413

[35] Danecek P, Bonfield JK, Liddle J et al Twelve years of SAMtools and BCFtools. Gigascience 2021; 10: giab008. 10.1093/gigascience/giab008 33590861 PMC7931819

[36] Kurtz S, Phillippy A, Delcher AL et al Versatile and open software for comparing large genomes. Genome Biol 2004; 5: R12. 10.1186/gb-2004-5-2-r12 14759262 PMC395750

[37] Marçais G, Delcher AL, Phillippy AM et al MUMmer4: a fast and versatile genome alignment system. PLoS Comput Biol 2018; 14: e1005944. 10.1371/journal.pcbi.1005944 29373581 PMC5802927

[38] Cingolani P, Platts A, Wang le L et al A program for annotating and predicting the effects of single nucleotide polymorphisms, SnpEff: SNPs in the genome of Drosophila melanogaster strain w1118; iso-2; iso-3. Fly (Austin) 2012; 6: 80–92. 10.4161/fly.19695 22728672 PMC3679285

[39] Seemann T . Prokka: rapid prokaryotic genome annotation. Bioinformatics 2014; 30: 2068–9. 10.1093/bioinformatics/btu153 24642063

[40] Khelik K, Lagesen K, Sandve GK et al NucDiff: in-depth characterization and annotation of differences between two sets of DNA sequences. BMC Bioinformatics 2017; 18: 338. 10.1186/s12859-017-1748-z 28701187 PMC5508607

[41] Mulvey MR, Haraoui LP, Longtin Y. Multiple variants of *Klebsiella pneumoniae* producing carbapenemase in one patient. N Engl J Med 2016; 375: 2408–10. 10.1056/NEJMc1511360 27974041

[42] Ghalayini M, Launay A, Bridier-Nahmias A et al Evolution of a dominant natural isolate of *Escherichia coli* in the human gut over the course of a year suggests a neutral evolution with reduced effective population size. Appl Environ Microbiol 2018; 84: e02377-17. 10.1128/aem.02377-17 29305507 PMC5835743

[43] Reeves PR, Liu B, Zhou Z et al Rates of mutation and host transmission for an *Escherichia coli* clone over 3 years. PLoS One 2011; 6: e26907. 10.1371/journal.pone.0026907 22046404 PMC3203180

[44] Jousset AB, Bonnin RA, Rosinski-Chupin I et al A 4.5-year within-patient evolution of a colistin-resistant *Klebsiella pneumoniae* carbapenemase-producing *K. pneumoniae* sequence type 258. Clin Infect Dis 2018; 67: 1388-94. 10.1093/cid/ciy293 29688339

[45] U.S. Food and Drug Administration . *Gepotidacin oral products*. 2025. https://www.fda.gov/drugs/development-resources/gepotidacin-oral-products

[46] Mendes RE, Kimbrough JH, Arends SJR et al In vitro activity of gepotidacin against *Escherichia coli* causing urinary tract infections between 2019 and 2021 in Europe, Russia, Israel, and Turkey, including molecularly characterized fluoroquinolone-resistant subsets. 33rd European Congress of Clinical Microbiology and Infectious Diseases (ECCMID). Copenhagen, Denmark, 2023. European Society of Clinical Microbiology and Infectious Diseases (ESCMID).

[47] Mendes RE, Hubler C, Kimbrough JH et al Activity of gepotidacin against *Klebsiella pneumoniae*, including molecularly characterized fluoroquinolone not susceptible subsets causing urinary tract infections in Europe and adjacent regions (2019–2022). 34th European Congress of Clinical Microbiology and Infectious Diseases (ECCMID), Barcelona, Spain, 2024. European Society of Clinical Microbiology and Infectious Diseases (ESCMID).

[48] Mendes RE, Kockler Z, Kapoor R et al Gepotidacin activity against *Escherichia coli* and *Klebsiella pneumoniae*, including molecularly characterized fluoroquinolone not susceptible subsets causing urinary tract infections in Europe and adjacent regions (2023). 39th Congress of the European Society of Clinical Microbiology and Infectious Diseases (ESCMID). Vienna, Austria, 2025. European Society of Clinical Microbiology and Infectious Diseases (ESCMID).

[49] VanScoy BD, Lakota EA, Conde H et al Gepotidacin pharmacokinetics-pharmacodynamics against *Escherichia coli* in the one-compartment and hollow-fiber *in vitro* infection model systems. Antimicrob Agents Chemother 2021; 65: e0012221. 10.1128/aac.00122-21 34543096 PMC8597728

[50] Arends SJR, West J, Thompson J et al *Frequency of spontaneous resistance for gepotidacin and levofloxacin against a collection of gram-negative and gram-positive organisms via single-step mutational analysis*. ASM Microbe, Washington, DC, 2022. American Society for Microbiology (ASM).

