# Supplementary material for: Characterization of isolates from two phase 3 uncomplicated urinary tract infection trials (EAGLE-2 and EAGLE-3) that show an apparent ≥4-fold increase in gepotidacin MIC between baseline and post-baseline visits
Source: JAC Antimicrob Resist. 2026 Sep 1;8(5):dlag186. doi: 10.1093/jacamr/dlag186 (PMC13531378; doi:10.1093/jacamr/dlag186)
Supplement: Supplementary file 1 [file dlag186_supplementary_data.docx]

# Supplemental Data

**Table S1.** Molecular characteristics of post-baseline isolates from patient #7

| **Uropathogen: *E. coli* Patient #7** | **Visit** | | |
| --- | --- | --- | --- |
|  | **Baseline** | **OT** | **TOC** |
| Colony count (CFU/mL) | ≥ 10^5^ | 10^4^ to < 10^5^ | ≥ 10^5^ |
| Gepotidacin MIC (µg/mL) | 16 | 128 | 128 |
| QRDR mutations | WT | ParC D79G | ParC D79G |
| AcrA expression (interpretation) | 5.02  (moderate expression) | 2.83  (similar to baseline) | 1.86  (similar to baseline) |
| MLST | 940 | 940 | 940 |
| Clinical symptom score | 2 | 0 | 0 |
| Days post baseline | N/A | 1 | 9 |
| SNPs observed | N/A | 2 | 4 |
| All isolates (baseline and post-baseline) had the following characteristics: Phenotypes = FQ-R/SXT-R, ESBL, MDR; ß-lactamase genes = CTX-M-15, EC-18-like; MLST = 940; O:H type = O-type nontypeable:H4.  An additional isolate outside the protocol-defined FU collection window, not described in this section, had a similar microbiological profile with the isolates from OT and TOC.  AcrA expression was determined as part of the Phase 3 uUTI clinical trials (EAGLE 2 and EAGLE 3) by real-time PCR reactions in the StepOne Plus instrument (Life Technologies, Foster City, CA, USA) using standard protocols.  Clinical symptom scores of 0 are defined as a clinical success (i.e. complete symptom resolution). Please reference primary manuscript for further information.^21^  CFU, colony-forming units; ESBL, extended-spectrum beta-lactamase-producing; FQ-R, fluoroquinolone resistant; FU, follow-up; N/A, not applicable; MDR, multidrug-resistant; MIC, minimum inhibitory concentration; MLST, multi-locus sequence type; OT, on-therapy; QRDR, quinolone resistance determining region; SNP, single nucleotide polymorphism; SXT-R, trimethoprim sulfamethoxazole resistant; TOC, test-of-cure; WT, wildtype; uUTI, uncomplicated urinary tract infection. | | | |
